# First case of autochthonous *Angiostrongylus vasorum* infection in a Norwegian dog

**DOI:** 10.1186/s13028-024-00765-7

**Published:** 2024-09-02

**Authors:** Julie Robbestad, Alejandro Jiménez-Meléndez, Lucy J. Robertson, Liva I. Vatne, Mari N. Hauback, Sivert Nerhagen

**Affiliations:** 1 Department of Companion Animal Clinical Sciences, Faculty of Veterinary Medicine, Norwegian University of Life Sciences, Post-Box 5003, N-1432 Ås, Norway; 2 Parasitology, Department of Paraclinical Sciences, Faculty of Veterinary Medicine, Norwegian University of Life Sciences, Post-Box 5003, N-1432 Ås, Norway

**Keywords:** Angiostrongylosis, Coagulopathy, Europe, Norway, Pulmonary hypertension, Regenerative anemia

## Abstract

A fifteen-month-old Pembroke Welsh corgi with respiratory distress, exercise intolerance, and moderate regenerative anemia was referred to The Norwegian University of Life Sciences, Small Animal Hospital.

Hematology revealed moderate regenerative anemia without evidence of hemolysis. Thoracic radiographs showed a generalized mixed interstitial to alveolar lung pattern and enlarged pulmonary arteries. Changes suggestive of moderate pulmonary hypertension were noted on echocardiography. Baermann fecal diagnostic flotation identified large numbers of *Angiostrongylus vasorum* larvae, and the AngioDetect serological antigen test was positive. The dog was treated with a two-week course with fenbendazole (51 mg/kg q24h po) and topical imidacloprid/moxidectin (250 mg/62.5 mg) and a one-week course with sildenafil (0.45 mg/kg q12h po). Complete clinical, clinicopathological and echocardiographic resolution was observed after only four weeks. Rapid improvement of echocardiographic abnormalities in cases with suspected pulmonary hypertension is not usually reported in cases with angiostrongylosis.

Infection with *A. vasorum* should be considered in dogs with respiratory signs and bleeding tendencies, even in countries with no endemic history or reported cases.

## Background

*Angiostrongylus vasorum*, also known as French heartworm, is a metastrongylid nematode for which several species of carnivores, especially canids, are the definitive hosts [1]. In dogs, infection with this parasite commonly leads to clinical signs from the respiratory tract, signs of hemostatic dysfunction, or neurological signs [2–5]. The spread of *A. vasorum* across Europe has been described as “explosive” [1], and different factors have been proposed as being relevant for this spread (e.g., climate change, increased dog movement, invasion of gastropods, fox urbanization) [1].

Norway has been one of the few European countries without confirmed cases of *A. vasorum* infection in domestic dogs with no travel history. In Scandinavia, the epidemiological situation of *A. vasorum* in canines and carnivores exhibits diverse patterns in different countries. Denmark has reported a high prevalence and recent spread of infection, particularly in certain regions, confirming that other wild carnivores (raccoon dogs, mink and polecats) in addition to red foxes, can act as reservoirs for cardiopulmonary nematodes in domestic animals. Furthermore, prevalences of *A.vasorum* in Danish red foxes have increased in most areas compared to previous studies, with areas like nothern Zealand detecting an increase from 48.7% in 1993 to 90% in 2002 [6, 7]. In Sweden, the endemic presence of *A. vasorum* has been confirmed, but the reported prevalence in both dogs and foxes is relatively low. In a nationwide seroepidemiological survey in Sweden, 0.10% dogs were positive for both antigen and antibodies, while the annual prevalence of positive faecal dog samples and of necropsied *A.vasorum* positive foxes varied from 0.3 to 0.9% and 0.0 to 1.4%, respectively [7]. Like Norway, Finland has a largely unknown geographical distribution of *A. vasorum*, but apparently autochthonous infection was reported in three dogs in 2019 [8]. Most of the countries with no reported endemic infections in dogs are situated along Europe’s eastern edge, such as Belarus, Latvia, Lithuania and Ukraine [1]; this may reflect lack of testing rather than absence of the parasite. In Norway, the first cases of *A. vasorum* infection were reported in 2016 in two foxes, geographically distant from each other (around 460 km distance). This was part of a surveillance program in which fecal samples from 234 foxes were examined by Baermann analysis, with positive samples verified by PCR and sequencing [9]. Later surveillance reported detection of *A. vasorum* antigen (Canine *A. vasorum* antigen test kit”; Angio Detect Test, IDEXX Laboratories) in four out of 67 fox blood samples in 2018 [10] and in eight out of 300 fox serum samples in 2019 [11].

While there are sporadic reports of diagnosis of *A. vasorum* in Norwegian dogs, these have been associated with dogs that have traveled to endemic areas. However, annual surveys of parasites among dogs imported into Norway during 2017 and 2018 found no cases of *A. vasorum* infection among 72 tested dogs imported during 2017 [12] or among 41 tested dogs imported during 2018 [13].

Pulmonary hypertension (PH) is frequently reported in dogs with angiostrongylosis [4, 14–17], but longitudinal studies assessing resolution of cardiopulmonary changes are lacking. In this case report, we describe the first case of canine infection with *A. vasorum* in a dog that had not travelled outside of Norway, with emphasis on clinical presentation and diagnostic investigations.

### Case presentation

A 15-month-old, female neutered Pembroke Welsh corgi was referred to The Norwegian University of Life Sciences, Small Animal Hospital with a history of a moderate regenerative anemia and respiratory distress. The dog was reported to have gradually worsening exercise intolerance, panting, weight loss, and lethargy over the previous three months. No cough was reported. The dog was up to date on vaccinations and had never traveled outside Norway.

On presentation, physical examination revealed a body condition score of 3/9, mild muscle loss, pale mucous membranes, tachycardia, tachypnoea, and a mildly increased mixed respiratory pattern, but was otherwise unremarkable. Investigations identified moderate to marked, regenerative anemia (PCV 19%, ref. 35–55%), in-saline agglutination was negative, and blood smear revealed no spherocytes or ghost cells. Serum biochemistry was unremarkable except for a moderate increase in C-reactive protein (45 mg/L, ref. 0–15 mg/L). Urinalysis was unremarkable.

Activated partial thromboplastin time (aPTT) and prothrombin time (PT) were prolonged (aPTT was 158.0 s, ref. 72.0–102 s and PT 18.0 s, ref. 11.0–17.0 s). Thromboelastography revealed low median angle (37.7 mm, ref. 42.9–67.9 mm) [18], LY30 and LY60 were not obtained due to technological malfunction.

Four-view digital thoracic radiographs revealed a diffuse to patchy, relatively symmetrical interstitial lung pattern with multifocal to peripheral alveolar components and a mild bronchial pattern. The caudal peripheral pulmonary arteries were moderately enlarged.

Abdominal ultrasonography showed diffusely heterogenous echotexture of the spleen. The study was otherwise unremarkable. Splenic fine-needle aspirates were performed without complications. Cytological examination was compatible with extramedullary hematopoiesis and reactive hyperplasia.

A complete transthoracic echocardiographic examination was performed under gentle restraint, without sedation, in right and left lateral recumbency using a General Electrics Vivid E95 ultrasound machine and a phased array transducer, 6S (Table 1). The echocardiographic examinations and measurements were performed by a single observer (LIV). Images were acquired according to published guidelines from right parasternal, subcostal, left apical and left parasternal windows [19]. Measurements of the right heart chambers were performed as previously described [20–22]. Measurements of the right pulmonary artery were performed by two-dimensional echocardiography in systole and diastole [23].

**Table 1:** Complete transthoracic echocardiographic examination pre- and post-treatment

Parameter evaluated	Pre-treatment	Post treatment	Unit	Reference intervals or range	Cut off predicting TRPG 50 mmHg > 50 mmHg	Reference
Body weight	11.4	11	Kg			
BSA (body surface area)	0.51	0.50	m^2^			
RPA systolic (right pulmonary arterial diameter in systole)	12.5	7.8	mm			
RPA diastolic (right pulmonary arterial diameter in diastole)	10	4.6	mm			
RPAD (RPA distensibility index)	20	41	%		< 29.1	[23]
MPA (main pulmonary arterial diameter)	16	10,1	mm			
Ao (Aortic diameter)	12.7	12.5	mm			
MPA/Ao	1.26	0.81			> 1.04	[23]
PA AT (pulmonary arterial accelleration time)	88	103	Ms		< 53.9	[23]
PA ET (pulmonary arterial ejection time)	204	193	Ms			
PA AT:ET	0,43	0.53			< 0.3	[23]
RVAD (right ventricular area in diastole)	5.3	2.9	cm^2^			
RVADN (RVAD normalised)	1.08	0.60	cm^2^/kg^0.655^	< 1.4		[22]
RVADI (RVAD normalised to body surface area)	10.36	5.81	cm^2^/m^2^	2.8–11.6 (range)		[21]
RVAS (right ventricular area in systole)	3.3	1.3	cm^2^			
RVASN (RVAS normalised)	0.61	0.25	cm^2^/kg^0.695^	< 0.8		[22]
FAC (fractional area change)	38	55	%	> 30%		[22]
RAA (right atrial area)	5.3	2.4	cm^2^			
RAAN (right atrial area normalised to body surface area)	10.4	4.8	cm^2^/m^2^	4.2–10.2 (range)		[21]
RVWd (right ventricular free wall)	5.5	4.8	Mm			
IVS flattening	yes	no				

Comparison of echocardiographic findings pre-treatment and post-treatment. Values exceeding established reference intervals or ranges are highlighted

**Fig. 1:**
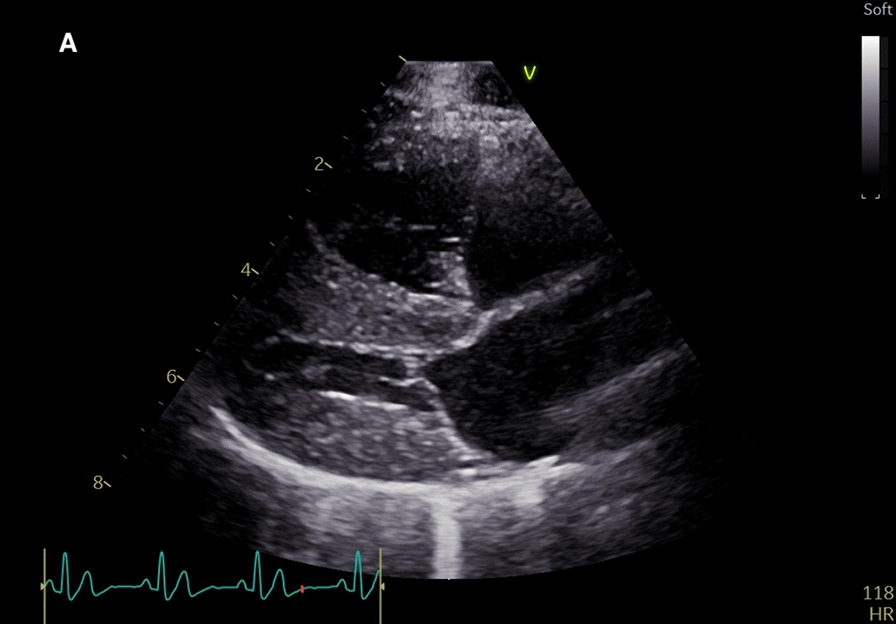
Echocardiography performed pre-treatment (**A**) from the right parasternal long axis four chamber view showing mild right chamber dilation, and from the short axis view showing mild interventricular septal flattening

**Fig. 2:**
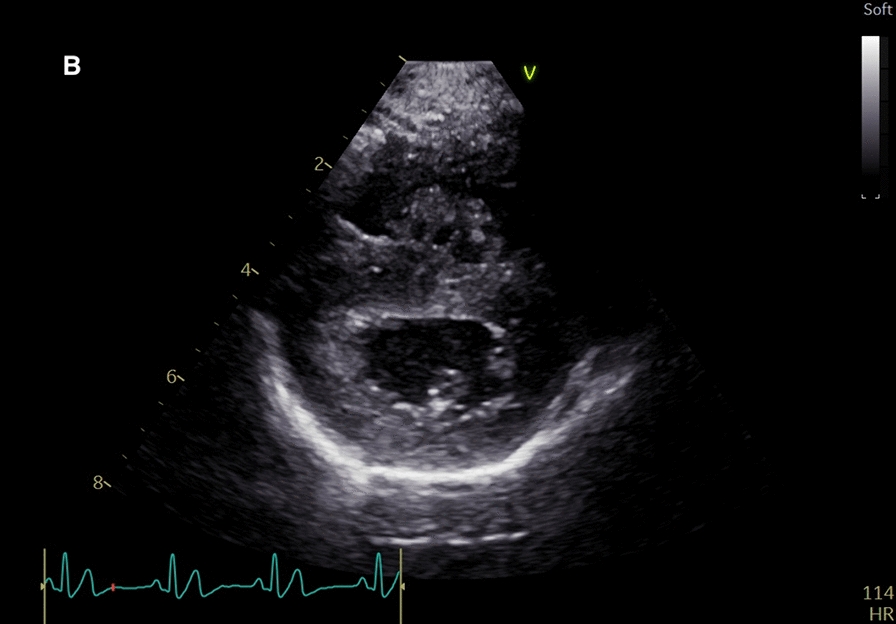
Echocardiography performed pre-treatment (**B**) from the right parasternal long axis four chamber view showing mild right chamber dilation, and from the short axis view showing mild interventricular septal flattening

The initial echocardiographic examination revealed mild dilation of the right ventricle when visually compared with the left ventricle (Fig. 1) [20, 22]. Interventricular septal flattening was noted (Fig. 2). The right atrium was mildly dilated with right atrial area index exceeding the published reference interval [21]. The main pulmonary artery was dilated when compared with the aorta, and the right pulmonary artery branch was dilated with reduced distensibility index [23]. The pulmonary arterial velocity profile revealed normal velocity, but the acceleration time was shorter than deceleration time [24]. Color flow Doppler did not reveal pulmonary insufficiency. Tricuspid regurgitation was seen using color flow Doppler from both right parasternal and left apical views, but it was not possible to measure the regurgitant velocity by spectral Doppler as no clear regurgitant profile could be acquired. In conclusion, the echocardiographic findings were consistent with significant PH [20–25].

**Fig. 3:**
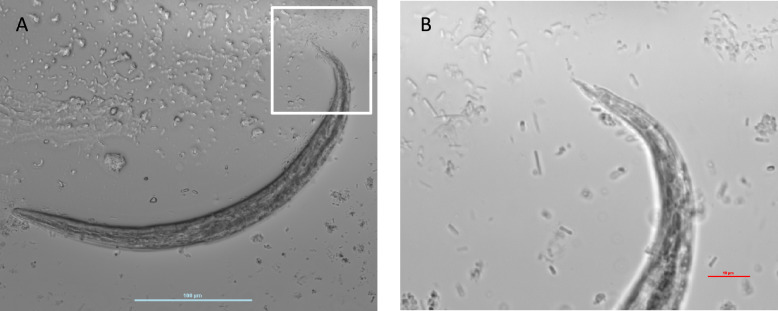
(**A**) *Angiostrongylus vasorum* first-stage larva (L1) visualized under light microscopy. (**B**) magnified view of the boxed area in Fig. 3A, including he distinctive kinky tail, observed at 40x magnification

An antigen test on serum for *Dirofilaria immitis* (SNAP 4DX, IDEXX Laboratories) was negative. Examination of a single fecal sample (25 g) by the Baermann method revealed numerous larvae of approximately 320 μm length with a characteristic tapered tail tip with a kink and dorsal spine (Fig. 3). Based on morphological characteristics, these were identified as first-stage larvae (L1) of *A. vasorum.* A strong positive result was obtained on a serum sample by Angio Detect Test (IDEXX Laboratories) as a confirmatory test.

The dog was treated with a packed red blood cell transfusion, oxygen cage with FI O2 40%, a two-week course with fenbendazole (51 mg/kg q24h PO [Panacur, MSD Animal Health, Intervet International B.V., Netherlands]), topical imidacloprid/moxidectin (250 mg/62.5 mg [Advocate, Bayer Animal Health GmbH, Germany]) and a one-week course with sildenafil (0.45 mg/kg q12 PO [Viagra, Orifarm]). Treatment with imidacloprid/moxidectin was repeated after two and four weeks. The tachypnoea, hyporexia, and lethargy improved significantly 24 h after initiation of treatment and the dog was discharged 36 h after initiation of treatment. Parasitological follow up was not performed.

At re-examination one week after discharge, the owner reported that the exercise intolerance had improved. Hematology, blood smear evaluation, and an echocardiographic examination were performed. The anemia had resolved (PCV 36%) and repeated echocardiographic evaluation revealed improvement in cardiac morphology, with persistent mild right ventricular eccentric hypertrophy however, without septal flattening and without dilatation of the pulmonary artery. Sildenafil treatment was terminated.

**Fig. 4:**
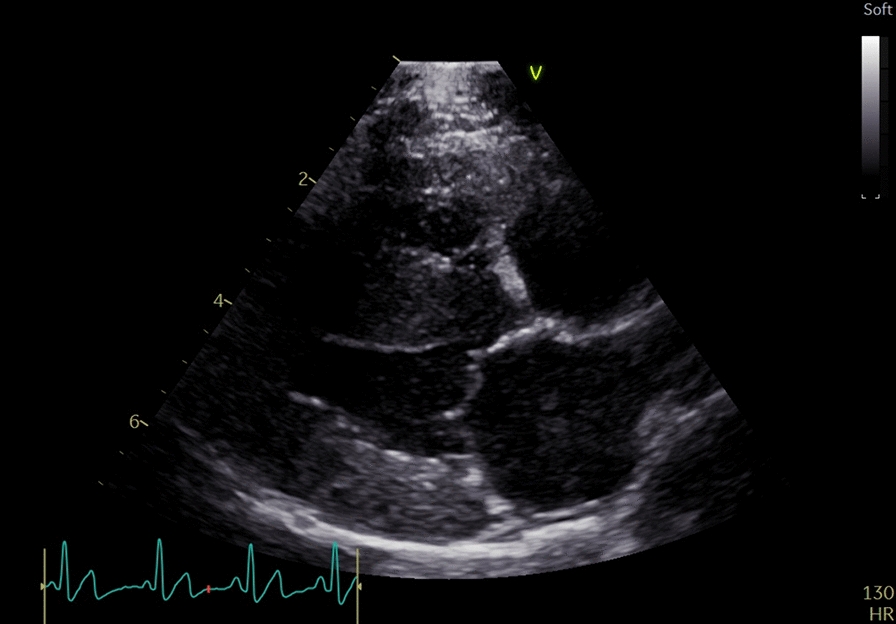
Echocardiography performed one month after commencement of treatment from the right parasternal long axis four chamber view showing normal cardiac morphology

Echocardiography was again repeated one month after discharge (Table 1). The right chambers were subjectively normal as illustrated in Fig. 4, and the right ventricular and—atrial area well within normal reference intervals [20–22]. The right pulmonary artery diameter was smaller than pre-treatment and the distensibility index was normal [23]. There was no evidence of tricuspid regurgitation. The pulmonary artery to aortic ratio was normal and the pulmonary acceleration to ejection time ratio was normal [24].

DNA was isolated from 5–10 larvae using DNeasy PowerSoil kit following the manufacturer’s recommendations, and PCR targeting the ITS-2 gene (forward primer sequence: CGA TGA CGG TAG CAA TGA CA; reverse primer sequence: TT GCG TGG TTC TTTACG TG) was run [26]. The reaction mixture (25 µL) consisted of 2 × Dream Green Hot start polymerase master mix (12.5 µL) 100 pmol of each primer and 4 µl of DNA template. PCR cycling conditions were as follows: 95 °C for 5 min followed by 35 cycles that included heating at 95 °C for 45 s, annealing at 58 °C for 45 s and an elongation step of 72 °C for 45 s followed by a final elongation step at 72 °C for 10 min. After confirmation on a 1.5% agarose gel, positive PCR products (218 base pairs) were purified with ExoSap-it Expres (ThermoFisher Scientific) according to the manufacturer’s instructions and Sanger-sequenced on both strands at a commercial facility. The sequences contained considerable allelic sequence heterozygosity; following sequence handling in BioEdit [27] and searching on NCBI Blast (BLAST: Basic Local Alignment Search Tool (nih.gov)), the highest similarity was found with a sequence of *A. vasorum* from Austria (98.28% identity) [28]. There were no available sequences in GenBank from previous findings in Norway, nor from Sweden or Finland. *A. vasorum* sequences obtained from Danish findings were at other gene targets. Sequences from our case have been submitted to GenBank (GenBank Accession number PP836527).

## Discussion and conclusions

*Angiostrongylus vasorum* has been described to cause bleeding tendencies in infected dogs [29] seen in approximately one-third of the cases [30, 31]. The pathophysiological mechanisms are not fully understood, and several hypotheses such as disseminated intravascular coagulation (DIC), coagulation deficiencies, secretion of anticoagulants by the parasite, and hyperfibrinolysis have been suggested [2, 29–33]. The results from aPTT/PT and thromboelastography analyses in this case were consistent with previous reports of a hypocoagulopatic state. Hyperfibrinolysis could not be ruled out [30, 33, 35], as percentage clot lysis time (LY30 and LY60) were not obtained, nor was tissue plasma activator added to the TEG [34]. We suspect the anemia seen in this dog was caused by pulmonary hemorrhage based on history, clinical signs and radiographic findings. Severe lung hemorrhage leading to death has been described in a previous case [36], and findings of diffuse hemorrhagic consolidation of infected lungs have been reported in post-mortem studies [37].

The clinical signs in this case could also be explained, in part, by the presence of PH. Clinically significant PH has been described in dogs naturally infected with *A. vasorum* [4]*,* however, only mild PH was reported in 2/6 experimentally inoculated dogs [15]. The cause of this difference may be explained by individual variability in the immunologic response to the parasite, an individual predisposition towards thrombosis and/or vascular reactivity and potential recruitment of intrapulmonary arteriovenous anastomoses [38, 39]. Pulmonary imaging findings suggestive of angiostrongylosis and PH previously reported are similar to the abnormalities noted on the thoracic radiographs in this case [39–41]. However, a notable finding on the thoracic radiographs in our case was the enlargement of the caudal pulmonary arteries. This finding has more commonly been reported in canine heartworm disease [41] but may also be seen in cases of angiostrongylosis [41, 42]. Hence, a mixed pulmonary pattern with a peripheral alveolar component with concurrent vascular changes should alert the clinician to consider *A. vasorum* as an important differential diagnosis to canine heartworm infection also in countries without an endemic presence of *A. vasorum.*

Interestingly, the echocardiographic changes seen in the dog in this report rapidly improved following therapy. Such rapid improvement has not been commonly reported. Previous studies of infected dogs with PH reported that despite normalization of clinical signs within two months of diagnosis, dogs with moderate to severe PH had persistent echocardiographic and radiographic changes [43]. Development of PH after treatment and apparent clearance of the infection has been described in another dog [44]. However, improvement of cardiac morphology following diagnosis has been reported in four dogs. Three dogs had rapid normalization of echocardiographic and radiographic abnormalities within two and three weeks [42], with the fourth reported to have complete reverse cardiac remodeling four months after diagnosis [45]. The causes of the individual differences seen in dogs developing PH and cardiac remodeling following angiostrongylosis is not known. Factors such as worm burden, longevity of infection and age of the animal may play a role, and studies are needed to characterize this further.

In our case, the dog was treated with the only available formulation, moxidectin, and imidacloprid spot-on ([Advocate, Bayer Animal Health GmbH, Germany]). Moxidectin was administered in conjunction with fenbendazole in an effort to maximize efficacy, as single therapy does not always fully clear the infection [46]. Unfortunately, despite owner encouragement, no post-treatment fecal sample was obtained. Therefore, the parasitological healing was not assessed, but it was considered as unnecessary based on the clinical healing and improvement of the patient.

It would have been relevant to have compared our molecular findings with those from previous cases of *A. vasorum* reported from Norway in foxes to determine relatedness; we urge colleagues to share their molecular findings on GenBank to enable geographic spread to be better monitored.

Here we describe the first case of *A. vasorum* infection in a Norwegian dog without a travel history. Although angiostrongylosis is a common diagnosis in dogs worldwide, this report is a reminder that *A. vasorum* is an important differential diagnosis in dogs with respiratory signs, bleeding tendencies or exercise intolerance, even in countries without current endemic presence of the parasite in dogs.

## Data Availability

The datasets used and/or analyzed during the current study are available from the corresponding author on reasonable request.

## Abbreviations

PH: Pulmonary hypertension
TEG: Thromboelastography
